# PATCH TESTING WITH DERMATOPHAGOIDES AND ITS CORRELATION WITH CHRONIC ECZEMA AND ATOPIC DERMATITIS

**DOI:** 10.4103/0019-5154.55633

**Published:** 2009

**Authors:** Chetna Kapur, Shrutakirthi D Shenoi, Smitha S Prabhu, C Balachandran

**Affiliations:** *From the Department of Skin and STD, Kasturba Medical College, Manipal, India.*

**Keywords:** *Atopic dermatitis*, *contact sensitization*, *dermatophagoides*, *eczema*, *patch test*

## Abstract

**Background:**

Chronic eczema is commonly encountered in the Indian set up. So also is atopic dermatitis. House dust mites (Dermatophagoides) are implicated in various diseases like atopic dermatitis, asthma, and perennial rhinitis. It has also been proven that patch testing with *Dermatophagoides pteronyssinus* (DP) is important for detection of contact sensitization in chronic dermatitis.

**Aims:**

To study clinical characteristics of DP mix positive patients with regards to chronic dermatitis and atopic dermatitis.

**Methods:**

Dermatology outpatients presenting to the department of Skin and STD of Kasturba Medical College (KMC), with clinically diagnosed atopic dermatitis and chronic eczema were chosen for the study. Inclusion and exclusion criteria were well demarked. Eighty six randomly selected patients of dermatitis were subjected to patch testing with standard series and DP mix.

**Results:**

Of the 86, 50 (58%) showed positive reaction to DP mix. Among these positive patients, chronic dermatitis was seen in 42 (84%) with involvement of exposed parts in 37 (74%). Atopic dermatitis was seen in 19 patients (38%) from DP positive group whereas it was observed in 4 patients (17%) from the other group.

**Conclusion:**

Dermatophagoides mix positivity was statistically significant in chronic eczema as well as atopic dermatitis. Patch testing is an important tool to detect delayed type allergy to house dust mite.

## Introduction

Chronic eczema is commonly encountered in the Indian set up. So also is atopy, especially in the southern states of Kerala and Karnataka. These patients often present with flare-ups which are difficult to explain with the present lifestyle. So, we aimed a study to determine whether house dust mite (HDM), which is ubiquitously present, has any role in exacerbation of these conditions. Though the role of dermatophagoides (DP) or HDM is well known in atopy, it is less studied in context with chronic eczema.

Dermatophagoides or HDMs are small, sightless, eight-legged arachnids, related to ticks, spiders, and scabies mites. These mostly reside in beddings, mattresses, carpets, and upholstered furnitures, in areas with low altitude and high humidity. The two common species include *D. pteronyssinus* and *D. farinae*.

House dust mite came into prominence as a pathogenic species, when in 1964 Voorhost *et al.*[1] suggested that most important source of house dust allergen was mite of genus *Dermatophagoides*. Platts mills *et al*.[2] were the first to observe a positive patch test (PT) to dust mite. Before 1950, atopic dermatitis (AD) was widely regarded as an allergic disease that could be caused by foods or inhalants. Tuft concluded from a series of experiments that among adults, HDM is the most important cause of AD. In AD, contact with aeroallergens (e.g., HDM, weeds, animal dander, and moulds) causes exacerbation in itching and skin lesions which can be investigated with epicutaneous application of these allergens by atopy patch test (APT). The diagnostic meaningfulness of PT with HDM allergens in dermatitis is still questionable.

In various studies, a positive PT to dust mite is associated with contact sensitization toward standard allergens too. We intended to correlate dust mite positive patients with AD and the enhanced responsiveness of DP mix positive patients to other contact allergens present in standard series.

### Aims

- To study the association between dust mite hypersensitivity and AD
- To study the association between positive PT to dust mites and contact hypersensitivity to other standard allergens

## Materials and Methods

**Figure 1:**
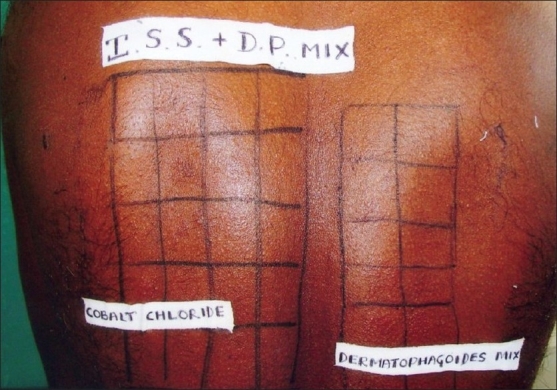
Marked back of the patient, patch tested with Indian Standard Series and dermatophagoides mix allergens after 72 hours

**Figure 2:**
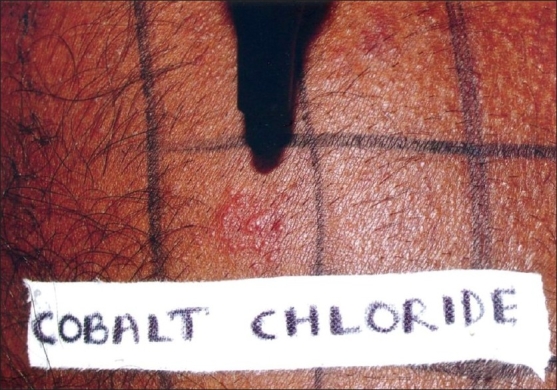
72-hour positive patch test reaction to cobalt chloride

**Figure 3:**
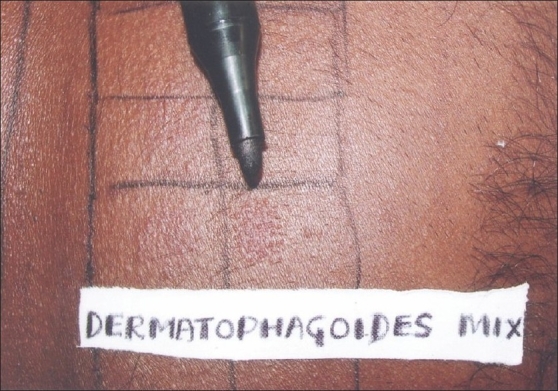
72-hour positive patch test reaction to dermatophagoides mix allergen

Patients with subacute or chronic dermatitis, aged 5-60 years, with body surface involvement of <50%, who presented to our department, were randomly selected. Diagnosis with regards to type of dermatitis was made on clinical grounds, visibly erythematous scaly patches and plaques, with or without oozing or crusting. AD was diagnosed clinically based on Haniffin and Rajka criteria – chronic or relapsing dermatitis, severe pruritus, personal or family history of atopy, typical morphology, the lesions being facial and extensor in infancy, thereafter mainly flexural pattern. Patients of active dermatitis with involvement of back or those on corticosteroids for last two weeks were excluded from the study. A total of 86 patients were initially included and patch testing with standard series along with DP mix was performed. DP mix was obtained from Chemotechnique Diagnostic, Sweden. The mix contained *D. pteronyssinus* and *D. pharinae* in the ratio of 50/50 in 20% petrolatum. Patch test was considered positive if positive readings were obtained at 48 and 72 hours, respectively [Figures 1–3]. Patients who tested negative for DP mix were later excluded from the study. Results were analyzed using ‘*Z*’ test for proportions to find *P*-value. From the DP mix positive patients, a detailed history including age at onset, duration, seasonal variations, symptoms, initial area involved, pattern of dermatitis, and specific aggravating factors was obtained. Personal and family history of atopy was also noted. The various parameters analyzed were age of patient, sex, duration of disease, clinical type of dermatitis, seasonal variation, family/personal history of atopy, first area involved, and PT positivity to standard allergen series.

## Observation and Results

A total of 86 patients were patch tested with DP mix and 50 patients tested positive. The following parameters were studied in those with positive PT results. The 36 patients who had negative test were excluded from the study.

Of the 50 patients, there were 28 males (56%) and 22 females (44%).The youngest patient was a seven-year-old boy and oldest was a 58-year-old male. The peak age at onset for either sex was in the third (30%) and fourth decades (28%). More than half the patients (58%) had disease duration between 1 and 10 years. The disease duration was less than a year in 26%, between 11 and 20 years in 10%, and greater than 20 years in 6%. Seasonal variation was observed in 10 patients (20%). Most of the patients reported the condition to deteriorate in winter season and others in rainy season. Most common aggravating factors detected were sunlight (10%), dust (10%), plants (10%), and detergents (8%).

### Clinical pattern of dermatitis

Out of 50 patients, 25 patients had clinical diagnosis of allergic contact dermatitis, ACD, (50%). Nineteen patients were diagnosed as AD (38%), three as irritant CD, two as air-borne CD, and one person had nummular eczema.

The areas first involved were feet (in 24% cases), followed by legs (20%), hands (18%), eyelids (12%), forearm (10%), flexures (8%), and face (8%).

Fifty percent of patients had lesions over exposed parts only, 32% had involvement of exposed and covered areas, and 18% had it in covered parts of the body.

### Personal and family history of atopy

Personal history of atopy was present in 21 (42%) and family history of atopy in 6 (12%) subjects, respectively. Six patients (17%) in the DP mix negative group had AD.

### Patch testing with standard series

Of the 50 patients who had positive treatment to DP mix, 42 (84%) patients showed positive treatment to other standard allergens also. Out of these 42, 19 (45%) had one other positive allergen, 14 (33%) had two other positive allergens, seven (17%) had three other positive allergens, and two (5%) had more than three positive allergens from standard series.

The most common allergen in standard series was fragrance mix in 8 (19%) followed by potassium dichromate, nickel, and formaldehyde in six (14%) each.

## Discussion

Atopic dermatitis is a chronically relapsing skin disorder frequently associated with elevated IgE level and history of atopy. Careful HDM avoidance regime have been associated with improvement in AD.[3] Various studies investigated the role of aeroallergens in pathogenesis of AD.[4] The gold standard for contact allergens is PT with the suspected allergen and can be employed to evaluate the role of dust mite in AD.[5]

In our study, AD was present in 38% of DP positive group as compared to 17% of negative group. A chi-square test was done to prove the statistical significance, which supports the role of dust mite in perpetuation of AD. As high as 84% of DP positive patients had reaction to other standard allergens, which is in accordance with study done earlier.[6] We also found that dermatitis in DP positive group was mostly chronic and presented over exposed parts.

The major allergens of HDM have been identified as cysteine proteases (group I allergens), serine proteases (group III allergens), and amylases (group IV allergens). These proteolytic enzymes are secreted by the digestive tract and are found in high concentration in mite fecal pellets. These pellets of the size of grass pollen grains (10-40 μm in diameter) readily become airborne and are easily inhaled.[2] Immediate hypersensitivity to allergens has been demonstrated in most mite allergic patients by skin testing, histamine release, and measurement of serum IgE antibodies, and these allergens are generally recognized as the major mite allergens.

### Relevance of dust mite allergy to diseases

Perennial rhinitis, asthma, and AD are thought to be exacerbated by dust mite allergens.

### Association of atopic dermatitis with mite allergen

Atopic dermatitis is an inflammatory skin disorder of relapsing course, which is associated with nonspecific hyperreactivity, hypersensitivity to environmental allergens, and immunological IgE production.

In 1982, Mitchell *et al*.[4] reported consistent eczematous reaction in some patients with AD following the application of a purified mite allergen directly to the skin for a prolonged period. Darsow *et al*. have documented a more specific role for aeroallergens in patients who have an air-exposed pattern of atopic eczema, emphasizing that all allergic contact reactions occur at the site of contact.[5] The reported success in the management of AD by eliminating dust mites supports a potential role of dust mites in the pathogenesis of AD. Some authors also found that patients who were dust mite test negative (intradermal or patch) also improved in a dust-free environment. This later finding supports the role of dust mite also acting as an irritant.

Bianca *et al*.[7] patch tested 313 AD patients and 100 healthy volunteers with DP and found 39% positive results in AD and 13% in healthy volunteers. A flare-up of dermatitis was found in atopic group by mite patch testing. Michel *et al*.[4] patch tested 355 nonrandomly selected AD patients and 398 normal controls with *D. pteronyssinus* antigen and demonstrated contact sensitization to mite in 20.8% in AD.

### Relevance of positive dermatophagoides mix patch test in atopics

Regardless of presence or absence of dermatitis, atopic individuals may acquire CD, which may be either irritant or allergic or combined, as atopy renders the skin vulnerable to nonspecific irritants, dust, or other strong allergens. The decreased irritancy threshold in atopics can be explained on the basis of conditioned hyperirritability localized to skin cells and increased amount of cytokines present in peripheral blood and skin that enhance the response to irritants. Due to this, the possibility of nonspecific irritant reaction to DP mix in atopics cannot be ruled out.

### Correlation with other standard allergens

Jochen *et al*.[6] studied DP mix patch testing in patients of CD and found that positive reaction to DP mix occurs preferentially in patients with a generalized enhanced responsiveness to contact allergen. They concluded that “some unidentified factors might contribute to positive reactions to DP mix that favor an enhanced general responsiveness to contact allergens.”

It may be concluded that a positive cutaneous reaction to mite material indicates an increased cutaneous responsiveness of skin to standard contact allergens. Patients with allergic skin diseases develop multiple sensitizations. Early detection and avoidance of sensitizing allergen is necessary.

### Allergic contact dermatitis in atopics

The incidence of ACD superimposed on atopy is still controversial. In our study, 15 (60%) out of 25 ACD and 15 (79%) out of 19 AD patients had positive reaction with standard allergens. AD patients had more positive results for standard PT allergens, as compared to ACD group. Erwin *et al*.[8] conducted PT with standard allergen series on atopics as well as nonatopics, wherein 77.2% of AD patients had positive PT as compared to 66.1% in nonatopic group. But, according to study done by Anton[9] out 214 atopics and 285 nonatopics, 37% of atopics had one positive reaction as compared to 52% in other group.

As the percentage of patients of AD with contact sensitivity varies from author to author, it may be agreed that patients with AD should be patch tested when indicated because they do develop contact allergy to a significant degree.

### Pattern of dermatitis

In our study, 50% of cases had lesions over the exposed parts and 32% had involvement of exposed as well as unexposed sites, whereas localized lesions in covered parts was seen in 18%. Darsow *et al*.[5] documented an air-exposed pattern in 17 out of 22 AD patients (77%) positive to dust mite. Imayama *et al*.[10] also noted higher incidence of positive results in patients whose eczema was predominantly on face, neck, and extremities than in patients with eczema of flexures.

## Conclusions

Our study concludes that house dust mites play an important role in patients with AD in the form of direct provocative antigens. However, as atopics have decreased irritancy threshold due to conditioned hyperirritability, a probability of false positive reaction is always present. A large percentage of AD patients (79%) had positive PT response to standard allergens too, in our study. The percentage of atopic patients with contact sensitivity varies from study to study. We agree that patients with AD should be patch tested when indicated.

Positive cutaneous reaction to mite allergen is usually coupled with increased cutaneous responsiveness to standard allergens. In our study, 84% of mite positive patients had positive reaction to at least one standard allergen. A significant percentage of clinically suspected ACD patients (60%) had positive reaction to dust mite. So, possibility of nonspecific irritant reaction cannot be ruled out. We propose further studies with larger sample size and healthy controls to prove these correlations.
